# Understanding CD4^+^ T cells in autoimmune bullous diseases

**DOI:** 10.3389/fimmu.2023.1161927

**Published:** 2023-04-17

**Authors:** A Yeong Lee, Taehee Kim, Jong Hoon Kim

**Affiliations:** Department of Dermatology and Cutaneous Biology Research Institute, Gangnam Severance Hospital, Yonsei University College of Medicine, Seoul, Republic of Korea

**Keywords:** autoimmune bullous disease, pemphigus, bullous pemphigoid, pathogenicity, CD4 T cells

## Abstract

Autoimmune bullous diseases (AIBDs) are a group of life-threatening blistering diseases caused by autoantibodies that target proteins in the skin and mucosa. Autoantibodies are the most important mediator in the pathogenesis of AIBDs, and various immune mechanisms contribute to the production of these pathogenic autoantibodies. Recently, significant progress has been made in understanding how CD4^+^ T cells drive autoantibody production in these diseases. Here, we review the critical role of CD4^+^ T cells in the production of pathogenic autoantibodies for the initiation and perpetuation of humoral response in AIBDs. To gain an in-depth understanding of CD4^+^ T-cell pathogenicity, antigen specificity, and mechanisms of immune tolerance, this review covers comprehensive mouse and human studies of pemphigus and bullous pemphigoid. Further exploration of pathogenic CD4^+^ T cells will potentially provide immune targets for improved treatment of AIBDs.

## 1 Introduction

Autoimmune bullous diseases (AIBDs) are a group of rare and serious skin diseases mediated by autoantibodies against adhesion molecules in the skin and/or mucous membrane, leading to the loss of cell-cell adhesion and blister formation. Autoantibodies produced by autoantigen-specific B cells cause blisters in AIBDs (1–3). However, reports suggests that CD4^+^ T cells are also involved in the production of pathogenic autoantibodies and play a critical role in the pathogenesis of AIBDs. It is not yet known how pathogenic CD4^+^ T cells are generated in AIBDs, but the studies of CD4^+^ T cells may provide clues to understanding the development of these diseases. This review describes current views on the role of CD4^+^ T cells in AIBDs, with a focus on pemphigus and bullous pemphigoid (BP).

## 2 Significance of CD4^+^ T cells in pemphigus

### 2.1 Clinical features of pemphigus

Pemphigus is a representative type of AIBDs mediated by autoantibodies targeting desmoglein (DSG) 1 and 3, which chronically cause blisters and erosions on the skin and mucous membrane (4). The etymology of pemphigus, which comes from the Greek *pemphix*, means bubble. Pemphigus most commonly occurs between the ages of 50 and 60 years, and consists of several forms, the main three being pemphigus vulgaris (PV), pemphigus foliaceus (PF), and paraneoplastic pemphigus (PNP) (3). PV usually begins as oral mucosal blisters, but PF shows superficial skin blisters without mucosal involvement. PNP is mostly associated with neoplasia and is characterized by extensive stomatitis and polymorphous skin eruptions, including blisters (5). The main histologic features are intraepidermal blisters with acantholysis as a consequence of the loss of adhesion between keratinocytes. Immunofluorescence studies are able to detect the IgG autoantibodies directed against the surface of keratinocytes. While the current cornerstone of treatment is systemic corticosteroid, high dosage and long-term usage are usually required for disease control, which can result in multiple adverse effects (6). In that reason, steroid-sparing agents such as mycophenolate mofetil and azathioprine are often combined (7). Further, intravenous immunoglobulin was found to reduce disease activity in pemphigus (8). Rituximab, a monoclonal depleting antibody that targets CD20 on B cells, shows partial or complete remission achieved 3 to 6 months after treatment in 75% of patients with pemphigus (9–11), and recent studies showed that early administration of rituximab shortens the time to remission (12). Rituximab is currently used as a first-line treatment in the U.S. and Europe (13).

### 2.2 Evidence of CD4^+^ T-cell pathogenicity in patients with pemphigus

**Figure 1:**
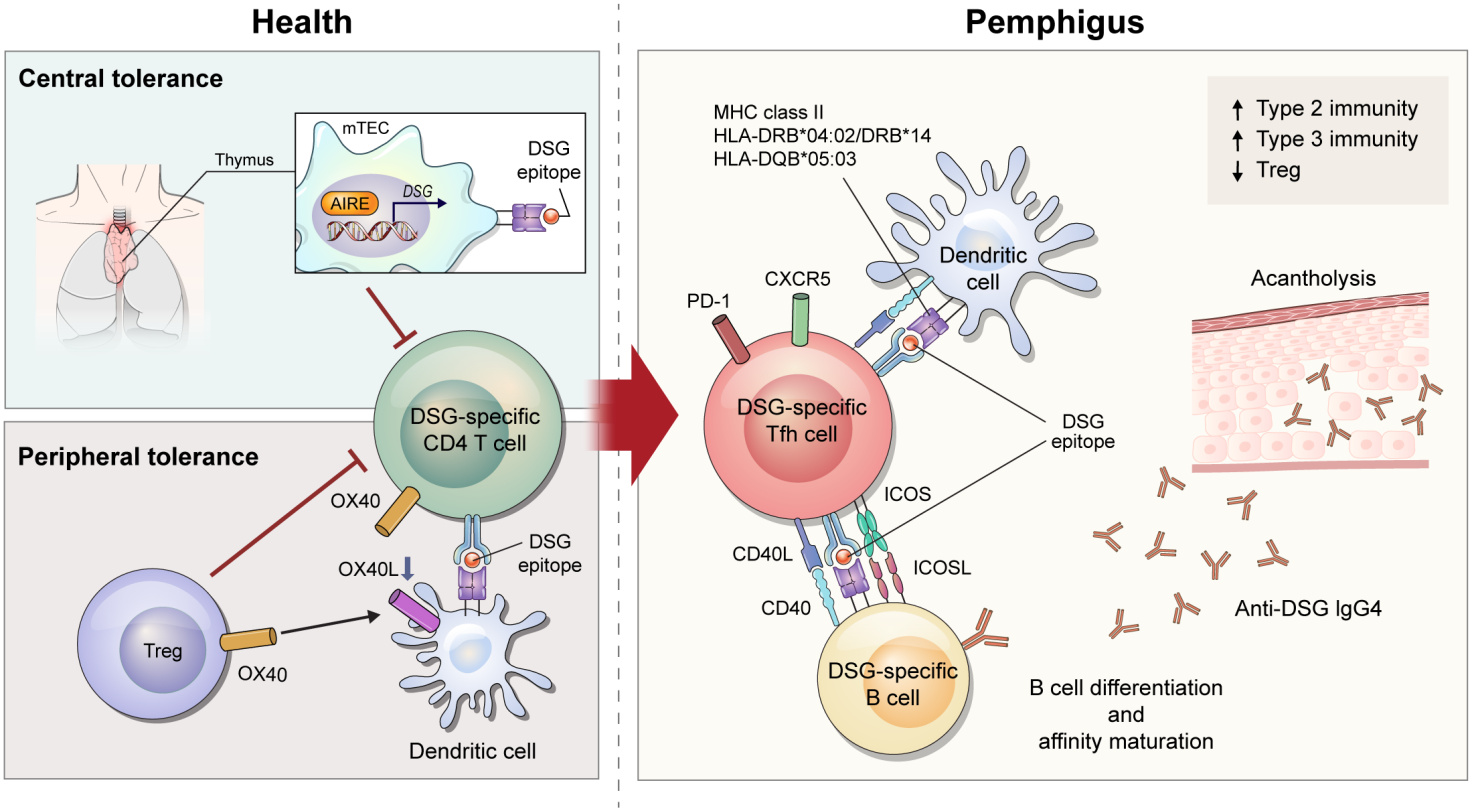
Schematic overview of immune mechanism of desmoglein (DSG)-specific CD4^+^ T cells in pemphigus. After recognizing DSG epitope expressed on major histocompatibility complex class II from medullary thymic epithelial cells under the control of AIRE, DSG-specific T cells undergo deletion in the thymus. When DSG-specific T cells escape from central tolerance, Tregs control tolerance in peripheral regions *via* OX40L-OX40 signaling. In a diseased state of pemphigus, tolerance is broken and DSG-specific CD4^+^ T cells become pathogenic. Of DSG-specific CD4^+^ T cells, ICOS^+^ Tfh cells interact with DSG-specific B cells to provide stimulatory signals including CD40L-CD40 interaction and cytokines. These signals lead to B-cell activation and proliferation, differentiation into plasma cells, and production of pathogenic DSG-specific IgG antibodies that induce acantholytic blister by loss of cell-cell adhesion in keratinocytes.

B cells are the most important cells in induction of pemphigus, but there is growing evidence that autoreactive CD4^+^ T cells play a pathogenic role. In particular, specific alleles of human leukocyte antigen (HLA) class II are associated with pemphigus. HLA-DRB1*04:02, HLA-DRB1*14 (14:01, 14:05, and 14:06), and HLA-DQB1*05:03 alleles are associated with PV, and HLA-DRB1*04 and HLA-DRB1*14 alleles are associated with PF and HLA-DRB1*01 alleles are especially associated with an endemic form of PF, also known as *fogo selvagem* (3, 14, 15). PNP is genetically associated with HLA-DRB1*03 (5, 16). These restricted types of HLA alleles differ among ethnic groups (17). DSG3-specific T-cell clones derived from patients with pemphigus vulgaris are proliferated following co-culture with a B-lymphoblastoid cell line expressing HLA-DRB1*14:01, HLA-DQB1*05:03, and HLA-DRB1*04:02 (18–20) (**Figure 1**). Although direct evidence for the production of pathogenic antibodies from DSG-specific B cells by these DSG-specific T cells is lacking, one study demonstrated that pathogenic anti-human DSG3 antibodies were induced by HLA-DRB1*04:02-restricted human DSG3-specific mouse T cells using humanized HLA-DRB1*04:02-transgenic mice (21).

The development of effective antibodies involves affinity maturation, a process by which B cells produce antibodies with higher affinity during subsequent responses to antigen. Affinity maturation is driven by somatic hypermutation and requires cognate-antigen interactions between B cells and CD4^+^ T cells for initiation. In one study, more anti-DSG3 antibodies undergoing somatic hypermutation were detected in patients with pemphigus vulgaris after diagnosis compared to before disease onset, and these antibodies exhibited increased binding affinity for DSG3 and induced better dissociation of keratinocytes (22). These data further support the idea that DSG-specific CD4^+^ T cells are pathogenic in pemphigus.

### 2.3 Pathogenicity, antigen specificity, and tolerance mechanism of CD4^+^ T cells in murine pemphigus models

Many studies have attempted to understand the pathogenicity of CD4^+^ T cells in pemphigus through use of active mouse models. These models are established by adoptive transfer of DSG3-immunized lymphocytes from *Dsg3*^–/–^ mice into DSG3-expressing *Rag1*^–/–^ or *Rag2*^–/–^ mice (23, 24). Immunization is accomplished by grafting DSG3-expressing wild-type mouse skin into *Dsg3*^–/–^ mice or by repeated injection of recombinant DSG3 protein into *Dsg3*^–/–^ mice (23, 24). Recipient *Rag1*^–/–^ or *Rag2*^–/–^ mice develop acantholytic blisters and pathogenic anti-DSG3 IgG, consistent with the features of PV. In this model, the PV phenotype is induced after adoptive transfer of both B and CD4^+^ T cells, but not after transfer of B cells alone (23). Specific subsets of pathogenic CD4^+^ T cells were well described in another study that divided CD4^+^ T cells into inducible costimulatory (ICOS)^+^ T follicular helper (Tfh) cells, ICOS^–^ Tfh cells, and non-Tfh cells. This study demonstrated that ICOS^+^ Tfh cells are required for the induction of the PV phenotype (24). Tfh cells are the major cells that directly interact with B cells, and ICOS is an important co-stimulator that maintains the phenotype of Tfh cells and promotes the migration of Tfh cells to the germinal center (25, 26). Indeed, ICOS^+^ Tfh cells have been found to be associated with B-cell differentiation in the PV mouse model (24) (**Figure 1**).

Since CD4^+^ T cells help B cells with cognate antigen interaction, it is necessary to investigate the antigen specificity of T cells to evaluate their role in pemphigus. T-cell receptor (TCR) transgenic mice (Dsg3H1 mice) have been used to understand the role of DSG3-specific CD4^+^ T cells in PV mouse models (27). After bone marrow transplantation from Dsg3H1 mice into *Dsg3*^–/–^ mice, DSG3-specific TCR-transgenic CD4^+^ T cells and *Dsg3*^–/–^ B cells were co-transferred into *Rag2*^–/–^ mice. In this model, DSG3-specific CD4^+^ T cells induce acantholysis, suggesting that DSG3-specific CD4^+^ T cells contribute to the generation of pathogenic antibodies in pemphigus. To analyze DSG3-specific CD4^+^ T cells in their physiologic state, another study generated a major histocompatibility complex (MHC) class II tetramer (I-A^b^/DSG3_516-530_) in the pemphigus mouse model (24). This study demonstrated that DSG3-specific ICOS^+^ Tfh cells captured by the tetramer were proliferating and associated with the production of anti-DSG3 antibody (24). (**Figure 1**)

While this mouse model exhibits an immunopathology similar to that of human pemphigus, it has limitations for deciphering the breakdown of tolerance in autoimmunity. Immune tolerance consists of central and peripheral tolerance. Central tolerance of T cells occurs as negative selection in the thymus, where autoreactive T cells are deleted by autoantigen-presenting medullary thymic epithelial cells (mTECs). mTECs can express tissue-specific antigens induced by transcription factors including autoimmune regulator (AIRE). AIRE induces expression of DSGs in mTEC (28), and T cells help B cells to produce anti-DSG3 IgG in *Aire*^–/–^ mice after adoptive transfer of *Dsg3*^–/–^ B cells (29) (**Figure 1**). Regulatory T cells (Tregs) are a key player in controlling peripheral tolerance. Anti-DSG3 antibody production decreases after transfer of Tregs in the active PV mouse model (30). In a recent study, DSG3-sepcific TCR-transgenic CD4^+^ T cells were able to overcome peripheral tolerance and induce dermatitis in depletion of regulatory T cell (DEREG) mice, but not wild-type mice. This study showed that OX40-OX40L signaling is important in disrupting Treg-dependent peripheral tolerance of DSG3-sepcific TCR-transgenic CD4^+^ T cells (31). (**Figure 1**) In this context, mechanisms of T-cell tolerance should be considered to understand the disease etiology of pemphigus.

### 2.4 Immunophenotype and function of CD4^+^ T cells in the human pemphigus

There are four subclasses of IgG (IgG1, IgG2, IgG3, and IgG4) in human, and IgG4 is the predominant subclass in patients with pemphigus (32). IgG4 is a well-known immunoglobulin that relies on a type 2 immune response. Thus, human T-cell studies have explored whether Th2 cells are involved in the pathogenesis of pemphigus. Early studies assessed the amount of DSG-reactive CD4^+^ T cells by stimulating DSG protein in peripheral blood mononuclear cells (PBMCs). IL-4-secreting DSG3-reactive Th2 cells predominate in the active phase of PV, whereas IFN-γ-secreting DSG3-reactive Th1 cells predominate in the chronic phase of PV and in healthy conditions (20, 33). When evaluating CD4^+^ T-cell subsets by observing expression of CXCR3 and CCR6, ICOS^+^PD-1^+^ circulating Tfh (cTfh) 2 cells, the Th2 phenotype of cTfh cells, were found to be associated with the production of autoantibodies against DSG3 in patients with PV (24). However, another study showed increased cTfh17 cells with responsiveness to DSGs in PBMCs from patients with pemphigus (34). In addition to an imbalance in conventional CD4^+^ T-cell subsets, the amount of circulating regulatory T cells were found to be decreased in patients with pemphigus. Moreover, FoxP3^+^CD25^hi^CD4^+^ T cells and DSG3-reactive IL-10^+^CD4^+^ T cells are reduced in blood from patients with pemphigus compared to healthy individuals (35, 36). Taken together, it is still unclear which subsets of CD4^+^ T cells play a key pathogenic role in human pemphigus and thus, this area require further study (**Figure 1**).

Although pemphigus is an autoimmune disease mediated by the systemic immune system, tertiary lymphoid structures resembling B-cell follicles are occasionally discovered in skin blisters of patients with pemphigus (37). In these TLSs, differentiated B cells, including DSG-specific B cells, are detected suggesting that these structures contribute to the pathogenicity of pemphigus (38). There are a number of T cells in TLSs, and IL-21^+^CD4^+^ T cells are expanded and produce IL-17 in lesions with TLSs (38). However, the role of various T-cell subsets in skin TLSs of pemphigus has not yet been fully studied and thus, represents an interesting area for future research.

### 2.5 Targeting CD4^+^ T cells in pemphigus

Many studies have been conducted to evaluate the efficacy of T-cell targeted therapy for the treatment of pemphigus. The CD40-CD40L interaction is essential for cognate antigen interaction of T and B cells. Indeed, anti-CD40L blocking antibody reduces disease induction in the PV mouse model (39). (**Figure 1**) In addition to the CD40-CD40L interaction, treatment of anti-ICOS blocking antibody *in vivo* also decreases disease progression (24). These therapeutic approaches can be used to control early disease provocation or prevent relapse, as these molecular interactions are required for disease initiation. Therefore, strategies to enhance the function of Tregs or expand Tregs can be applied to treat the active phase of the disease. Currently, adoptive polyclonal Treg cell therapy is being investigated in a phase I clinical trial for pemphigus (40).

## 3 Significance of CD4^+^ T cells in bullous pemphigoid

### 3.1 Clinical features of BP

Bullous pemphigoid (BP) is an AIBD characterized by autoantibodies against bullous pemphigoid antigens (BPAG) 1 and 2, and is distinguished from pemphigus in that blisters develop in the subepidermal layer (41, 42). The mean age of onset is older than that of pemphigus, and the relative risk of BP increases with age. Mortality in patients with BP is quite high (approximately 20% of the first-year mortality) and may be due to comorbidities or secondary infections (43). Typically, BP presents with multiple tense bulla in urticarial lesions of the skin. Mucosal involvement may occur, but not as often as it does in pemphigus vulgaris. Pruritus is usually present and can be intense. Some medications, such as immune checkpoint inhibitors and dipeptidyl peptidase-4 (DPP4) inhibitors, have been reported to be associated with the development of BP (44, 45). Histologic examination reveals subepidermal blisters usually accompanied by eosinophilic infiltration. IgG autoantibodies deposited along the basement membrane can be detected by direct immunofluorescence. In order to distinguish BP from other pemphigoids, salt-split skin is used in an indirect immunofluorescence study, looking for the presence of autoantibodies binding along the epidermal side of blister. First-line treatment options are topical and systemic corticosteroids (46, 47). Doxycycline, azathioprine, mycophenolate mofetil, or other immunosuppressive agents may be used as steroid-sparing agents (48). In severe cases, intravenous immunoglobulin, rituximab, omalizumab, and dupilumab can be administered (49–51).

### 3.2 Evidence of CD4^+^ T-cell pathogenicity in patients with BP

Although pathogenic autoreactive B cells undergoing affinity maturation have not been detected in patients with BP, there is indirect evidence that CD4^+^ T cells contribute to the development of autoantigen-specific B cells in BP. Epitope spreading refers to the intra- or inter-molecular diversification of antibody epitopes possibly caused by antigen cross-reactivity or cognate B-T cell interaction, and frequently occurs in autoantigen responses of BP (52, 53). These phenomena suggests that BPAG1- and BPAG2-specific B cells might experience somatic hypermutation *via* the help of BPAG1- or BPAG2-specific CD4^+^ T cells in BP. Further indirect evidence of this is the MHC class II restriction in patients with BP. Although the HLA genetic predisposition for BP exhibits a more diverse pattern across countries than does that of pemphigus, HLA-DQB1*03:01 is primarily associated with BP in many countries (54). These findings indicate that CD4^+^ T cells may be involved in the development of autoantibodies in BP.

### 3.3 Pathogenicity and tolerance mechanism of CD4^+^ T cells in murine BP models

**Figure 2:**
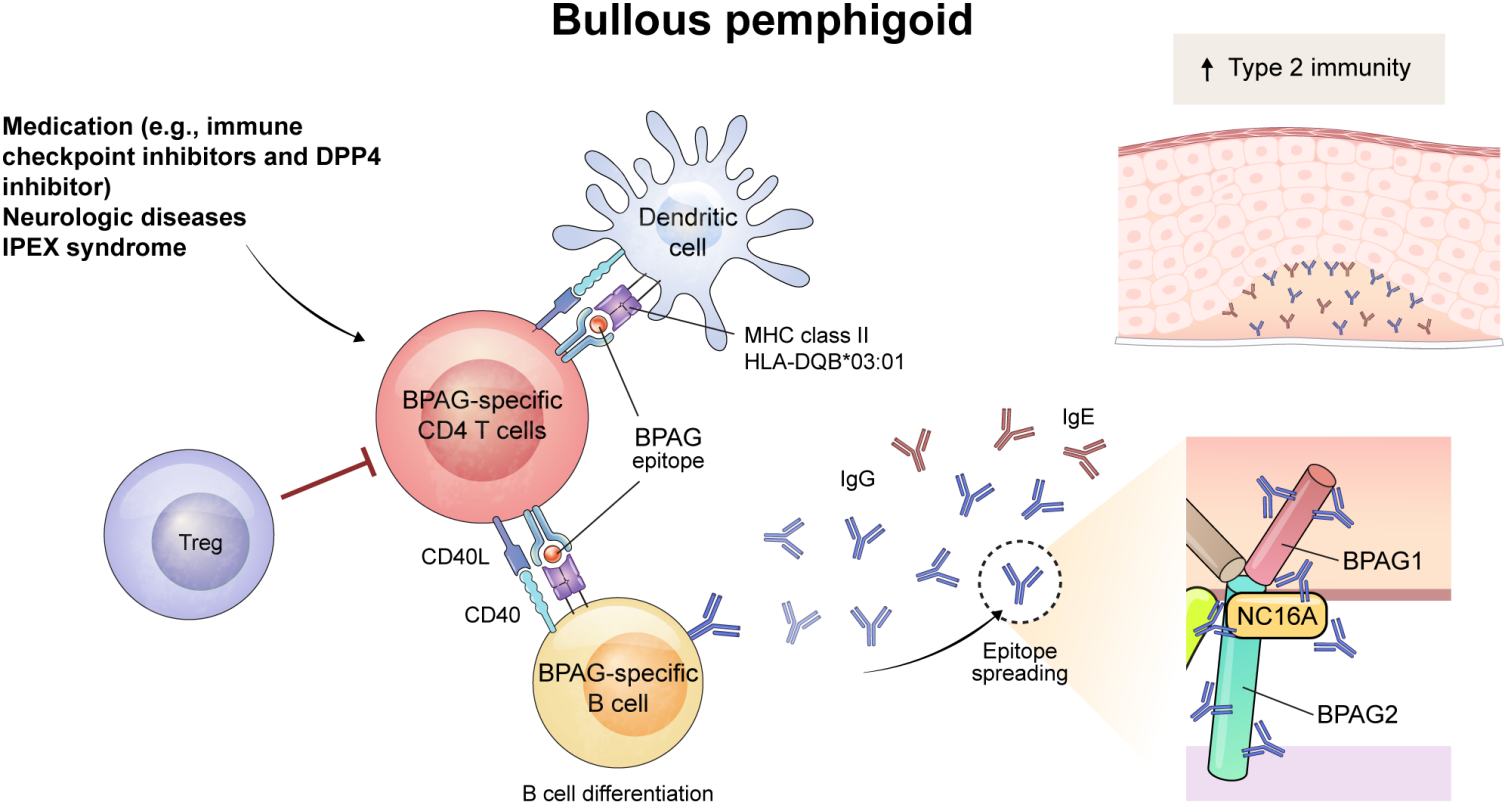
Schematic overview of immune mechanism of BPAG-specific CD4^+^ T cells in bullous pemphigoid (BP). The triggering factor for BP can be medication (e.g., immune checkpoint inhibitors and dipeptidyl peptidase-4 inhibitor) or neurologic diseases. The process begins with the presentation of a BPAG epitope by dendritic cells through MHC class II, including HLA-DQB*03:01, which activates BPAG-specific CD4^+^ T cells. Also, dysfunction of Treg cells play a role in the development of BP, as evidenced in patients with IPEX syndrome. This leads to interaction with BPAG-specific B cells through CD40-CD40L interaction, resulting in the differentiation of B cells and the epitope spreading of IgG and IgE autoantibodies. Among them, NC16A domain of BPAG2 is recognized as a major pathogenic epitope of autoantibodies. These autoantibodies bind to the basement membrane zone of the skin and mucous membranes, inducing subepidermal blistering in BP. The increased type 2 immunity was seen in BP and might contribute to the development of the diseases.

An active mouse model of BP was established using BPAG2-humanized *Rag2*^–/–^ mice (*Col17*^m–/–,h+^*Rag2*^–/–^) (55, 56). Similar to the active PV mouse model, splenocytes from wild-type mice immunized by grafting human BPAG2-expressing mouse skin are adoptively transferred into *Col17*^m–/–,h+^*Rag2*^–/–^ mice. These mice developed subepidermal blisters with deposition of anti-human BPAG2 antibody in the basement membrane zone. In this mouse model, the BP phenotype does not emerge after adoptive transfer of CD4^+^ T-cell depleted splenocytes (55). Whether these pathogenic CD4^+^ T cells directly help B cells and are specific for BPAG2 is not yet known, but the CD40-CD40L interaction is crucial for the early stages of the disease in the active BP mouse model (57). In addition, the intramolecular epitope spreading to NC16A domain of human BPAG2 is suppressed by blocking CD40L in the mouse model, suggesting that the cognate B-T interaction is responsible for epitope spreading to NC16A of BPAG2 (56) (**Figure 2**).

As seen in pemphigus, Tregs also are involved in the pathogenesis of BP. Interestingly, *Foxp3*-mutant scurfy mice spontaneously produce autoantibodies that target the basement membrane zone and contain pathogenic anti-BPAG1 autoantibodies that cause subepidermal blisters (58). In addition, when T-cell deficient nude mice were transferred with CD4^+^ T cells from scurfy mice, subepidermal blisters positive for autoantibodies developed (58). These results indicate that autoreactive CD4^+^ T cells are crucial to induce subepidermal blisters. These autoimmunity in scurfy mice is dependent on STAT6 pathway, a JAK-STAT signaling pathway known to be important in Th2 immunity, and Tfh cells are also decreased in the lymph nodes and spleen of *Stat6*^–/–^ scurfy mice (59) (**Figure 2**).

### 3.4 Immunophenotype and function of CD4^+^ T cells in the human BP

In patients with BP, the NC16A domain of BPAG2 protein is recognized as a major pathogenic epitope, and serum levels of anti-NC16A IgG correlate with clinical disease activity (60). Levels of serum anti-NC16A IgG positively correlates with the frequencies of PD-1^+^ and ICOS^+^ circulating Tfh cells and serum IL-21 level in patients with BP (61). Besides anti-NC16A IgG, anti-NC16A IgE is known to be associated with the pathogenicity and disease severity of BP (62, 63). Since IgE is typically mediated by Th2 cells, it has been thought that Th2 immunity is mainly involved in BP. Indeed, BPAG2-reactive Th1 cells can be detected in both healthy individuals and patients with BP, but BPAG2-reactive Th2 cells are only observed in patients with BP (64). Several studies have used epitopes in the NC16A domain to detect BPAG2-reactive CD4^+^ T cells in patients with BP (65–67), although the immunodominant epitopes of autoreactive CD4^+^ T cells in BP varies among individuals (68),

Whether the frequency of Tregs is altered in peripheral blood and skin from patients with BP is controversial (69–73). However, a substantial role of Tregs in the disease pathogenesis of human BP can be confirmed by observing patients with immune dysregulation, polyendocrinopathy, enteropathy, and X-linked syndrome (IPEX) syndrome (59, 74). IPEX syndrome is a genetic disorder manifesting an absence or dysfunction of Tregs due to *FOXP3* mutation. Several cases of IPEX syndrome show features of BP and circulating autoantibodies to BPAG1 and/or BPAG2, suggesting that Tregs contribute to the suppression of autoantibody production in BP (59, 74, 75). Human CD25^+^FoxP3^+^ Tregs are subdivided into three populations based on CD45RA and FoxP3 expression; CD45RA^+^FoxP^low^ naïve Treg, CD45RA^–^FoxP3^hi^ effector Treg, and CD45RA^–^FoxP3^low^ non-suppressive T cells (76). One study of these three subsets found that the frequency of effector Tregs correlated with disease severity in patients with conventional BP; however, in patients with BP induced by DPP4 inhibitors, Tregs did not correlate with disease severity (77). Therefore, the contribution of Tregs to disease pathogenesis may differ depending on the types of BP.

## 4 Conclusion

Although B-cell targeting therapies have been successfully used to treat AIBDs, curing the disease remains challenging. Therefore, many clinical trials are underway to develop more effective treatments for AIBDs (78). As shown in this review, a number of studies in mouse models and in humans have investigated the contribution of CD4^+^ T cells to the pathophysiology of AIBDs. However, the underlying regulatory mechanisms and their precise immunological roles have not yet been fully described. A deeper understanding of autoreactive CD4^+^ T cells and Tregs may support the identification of therapeutic targets to completely cure AIBDs. Furthermore, to develop optimal treatment strategies for AIBDs, it is necessary to elucidate the immune networks between antigen-presenting cells, autoreactive T and B cells, and Tregs through a comprehensive analysis.

## Author contributions

AL and TK wrote the manuscript. JK wrote and edited the manuscript. All authors contributed to the article and approved the submitted version.
